# Entailing the Next-Generation Sequencing and Metabolome for Sustainable Agriculture by Improving Plant Tolerance

**DOI:** 10.3390/ijms23020651

**Published:** 2022-01-07

**Authors:** Muhammad Furqan Ashraf, Dan Hou, Quaid Hussain, Muhammad Imran, Jialong Pei, Mohsin Ali, Aamar Shehzad, Muhammad Anwar, Ali Noman, Muhammad Waseem, Xinchun Lin

**Affiliations:** 1 State Key Laboratory of Subtropical Silviculture, Zhejiang A&F University, 666 Wusu Street, Lin’An, Hangzhou 311300, China; furqan2210uaf@zafu.edu.cn (M.F.A.); 20184007@zafu.edu.cn (D.H.); quaid_hussain@yahoo.com (Q.H.); 2019202011010@stu.zafu.edu.cn (J.P.); 2 Colleges of Agriculture and Horticulture, South China Agricultural University, Guangzhou 510642, China; muhammadimran@scau.edu.cn (M.I.); m.waseem.botanist@gmail.com (M.W.); 3 State Key Laboratory of Microbial Resources, Institute of Microbiology, Chinese Academy of Sciences, Beijing 100101, China; moh.uaf2356@outlook.com; 4 Maize Research Station, AARI, Faisalabad 38000, Pakistan; aamarshehzad1763@gmail.com; 5 Guangdong Technology Research Center for Marine Algal Bioengineering, Guangdong Key Laboratory of Plant Epigenetics, College of Life Sciences and Oceanography, Shenzhen University, Shenzhen 518055, China; anwar_uaar@yahoo.com; 6 Department of Botany, Government College University, Faisalabad 38000, Pakistan; alinoman@gcuf.edu.pk

**Keywords:** sustainable crop production, genome, next-generation sequencing (NGS), genetic resources, metabolomics, metabolites, stress tolerance, bamboo

## Abstract

Crop production is a serious challenge to provide food for the 10 billion individuals forecasted to live across the globe in 2050. The scientists’ emphasize establishing an equilibrium among diversity and quality of crops by enhancing yield to fulfill the increasing demand for food supply sustainably. The exploitation of genetic resources using genomics and metabolomics strategies can help generate resilient plants against stressors in the future. The innovation of the next-generation sequencing (NGS) strategies laid the foundation to unveil various plants’ genetic potential and help us to understand the domestication process to unmask the genetic potential among wild-type plants to utilize for crop improvement. Nowadays, NGS is generating massive genomic resources using wild-type and domesticated plants grown under normal and harsh environments to explore the stress regulatory factors and determine the key metabolites. Improved food nutritional value is also the key to eradicating malnutrition problems around the globe, which could be attained by employing the knowledge gained through NGS and metabolomics to achieve suitability in crop yield. Advanced technologies can further enhance our understanding in defining the strategy to obtain a specific phenotype of a crop. Integration among bioinformatic tools and molecular techniques, such as marker-assisted, QTLs mapping, creation of reference genome, de novo genome assembly, pan- and/or super-pan-genomes, etc., will boost breeding programs. The current article provides sequential progress in NGS technologies, a broad application of NGS, enhancement of genetic manipulation resources, and understanding the crop response to stress by producing plant metabolites. The NGS and metabolomics utilization in generating stress-tolerant plants/crops without deteriorating a natural ecosystem is considered a sustainable way to improve agriculture production. This highlighted knowledge also provides useful research that explores the suitable resources for agriculture sustainability.

## 1. Introduction

**Figure 1:**
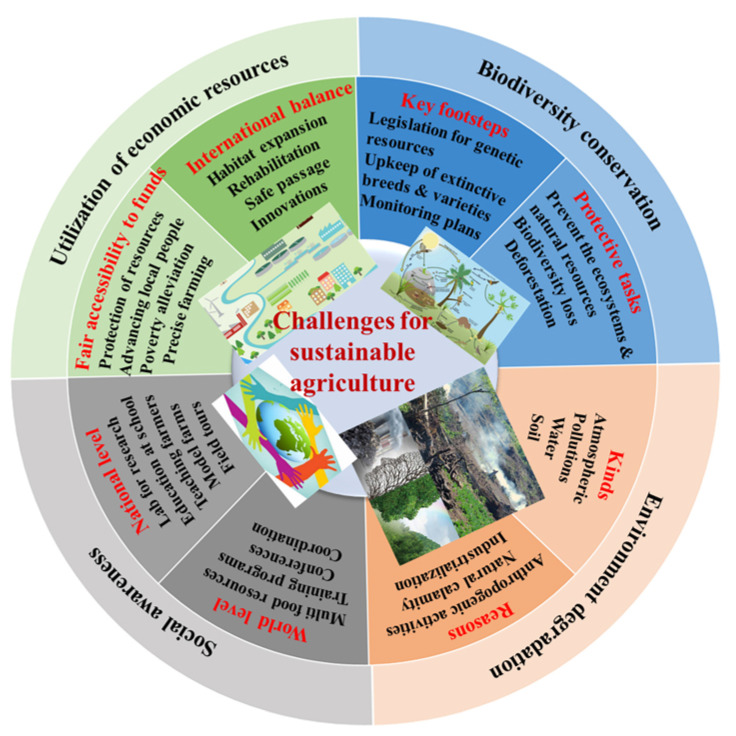
The key challenges for sustainable agriculture production. Four major challenges for agriculture and raising serious issues across the world.

The current global population is forecasted to cross ~9.8 billion in 2050 [1,2,3]. Several parts of the world are at risk of food insecurity [3]. After a consistent decline in crop production, the frequency of malnutrition in different world areas overturned the passage beginning in 2015 and has continued to climb. Malnutrition is predicted to increase up to 9.8% in 2030, presently soaring at ~9% worldwide, leading to a hunger crisis among ~850 million persons. Furthermore, agriculture production endures consuming a vast resource footmark, captivating ~38% of the surface area of the Earth and utilizing approximately 70% and 1.2% of fresh water and global energy resources, respectively, of the world [1,4]. Besides agriculture consumption, other challenges include the degradation of agricultural land, urbanization, increasing water shortage, and dependence on carbon-economy-based synthetic inputs [1,5]. Agriculture production should be increased more as compared to the current progress in an ecofriendly, sustainable, and safe way [6,7]. After that, the food supply can be maintained to deliver enough food worldwide and avoid food insecurity events. Different types of plants have been domesticated to use as a food source and confront the huger events across the globe. Still, environmental alterations and biotic stresses have been the off-putting reason for reaching the targeted, sustainable crop yield. For example, biotic (pests, microbes, etc.) and abiotic (temperature variations, incidents of drought, salinity, etc.) stressors adversely affect agriculture production [4,8,9,10]. Therefore, feeding such a huge population will be a serious test along with creating livelihood opportunities, limited resources, and various global challenges as aforementioned to gain sustainable agriculture production (Figure 1).

**Figure 2:**
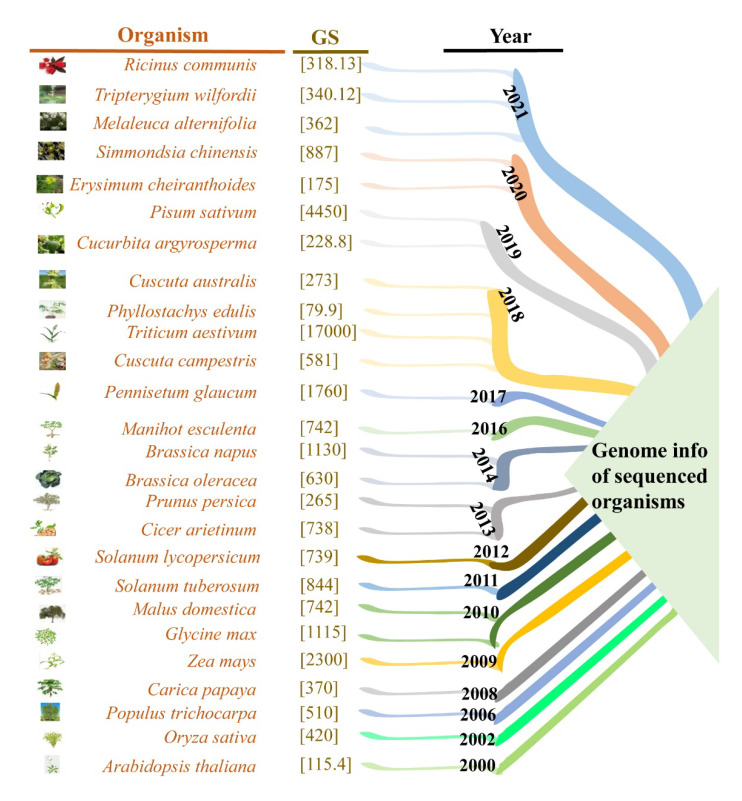
Timeline of the sequenced organisms. Sequencing technologies generated genomic information bank of various organisms and suggested an exciting research forum for further innovation. In the figure, genome size in mega-bases [Mb]-GS.

Therefore, humanity is under the threat of increasing world hunger, and the United Nations commission has set a goal, which is the Zero-Hunger Target by 2030. It is obligatory to achieve this goal by employing sustainable resources via safeguarding crop production in extreme environments while decreasing the resources indispensable to nourish a burgeoning global population. To entail a comprehensive system-centered technology that integrates innovative farming approaches, long-term sustainable agronomic practices, and value-added climate-resilient crops, genomic-based technologies offer, for this task, solid foundational tools and genetic tools insights for shaping the future agriculture [4]. The whole-genome sequence (WGS) of *Arabidopsis thaliana* was developed 21 years ago, and later on, rice was the first crop in 2002 with available WGS and so on [11,12,13,14,15] (Figure 2). 

Access to the sequenced genome of various plants has exploited the potential genomic targets for improving the agronomic traits in crops. Genetic manipulations for desirable variations permit crop production, improving flexibility against harsh environments and pathogen stressors, and resulting in the generation of novel types of a plant [16,17,18]. Moreover, genomic is vital for advances in the crop sciences to fulfill the agriculture demands. Strategies related to genome sequencing have been improved to offer knowledge for crop enhancements during the last century [16]. Now, WGS data of the complex/polyploidy crops can be generated using NGS strategies such as long-read single-molecule sequencing strategy. For example, the wheat genome (hexaploid) was generated through NGS [19,20,21,22]. It is the fruit of the advancement in technologies, setting the stage to obtain elaborative information (info) by performing the genome-based interpretation of epigenomic data, consisting of the 3-D validation of the nucleus genome, the huge metabolome, transcriptome, and proteome [23,24,25]. Robot-based technologies also help in gaining agriculture sustainability. For example, geosatellite imaging can forecast heatwave, drought, etc., events, and high-throughput phenology technologies and the involvement of drone technology have been used for planning a better strategy to improve crop production. Recent developments in the computational approaches to obtain detailed results about an individual or a big dataset by involving artificial intelligence are further strengthening our understanding of sustainable crop production [26,27,28].

For now, CRISPR/Cas9 technology is a valuable editing system to generate modifications in genes and manipulate new genomes with precision to explore unknown mechanisms and is also aimed at the de novo domestication of an important crop to produce a high yield and short breeding cycle, etc. [29,30]. Progress in the genomic techniques provides new dimensions and inspires prospects for crop improvement by implying the genomic resources in the upcoming years. Thus, the assimilation among various streams of congruent and/or inconsistent data is key to cultivating an innovative approach into crop science toward the practical application in agriculture. Furthermore, these technologies have a broad spectrum application in plant biology and other fields of life sciences, such as the biomedical field, and will possibly affect the future of agriculture. Therefore, formulating the recent advances related to the metabolome and sequencing approaches for sustainability in production is crucial. It is also required to obtain a better understanding of a crop and to define a strategy to solve the global issues associated with current and past agriculture.

## 2. Progress in Sequencing

DNA sequences store a bulk quantity of genetic information of life. The strategies that decode this genetic information can make a paradigm revolution in the multiscientific disciplines. Frederick Sanger in 1977 elaborated on the DNA sequencing technology (DNA-seq). DNA-seq was also called Sanger’s sequencing, which was established on the basis of a chain termination system [31]. More improvements were introduced by Maxam and Gilbert [32] using chemical amendments of DNA and following cleavage at a particular nucleotide base(s).

### 2.1. First-Generation Sequencing

Sanger’s sequencing displayed high productiveness and less radioactivity. Later, Sanger’s sequencing was termed first-generation sequencing (FGS). FGS was utilized at the commercial level in many fields [33]. Sanger’s method has been used to generate small and large sets of FGS data of organisms, such as bacteria and human, respectively [34,35,36]. Previously, sequencing was challenging and required radioactive reaction reagents. Other constraints were limited output data using a single reaction, laborious work required to sequence diploid/haploid DNA by performing subcloning. Subcloning produces a specific template of DNA for sequencing. Despite many efforts to improve FGS, the sequencing strategy had touched its ceiling due to time consumption and cost [37,38]. For example, in the past, 10 million US dollars was invested to create an additional genome of human [39]. The above-described limitations eventuated the exploration of new approaches for sequencing. 

In the past, the sequencing field had relied on the induction of the first automatic machine in 1987 by Applied Biosystems. This sequencing machine named AB370 contains capillary electrophoresis (CE). CE enhanced the speed of sequencing and accuracy of the AB370. The reported detection of nucleotides at one time and a day were 96 bases and 500 kilo bases, respectively, by AB370. Overall, AB370 can generate a read-length up to 600 bases. An upgraded model (AB373xl) can produce 900 base read-lengths by detecting 2.88 megabases per day since 1995. At that time, powerful sequencing machines were developed to save time and the cost of consumption [38]. FGS had been utilized to determine the expressed-sequence tags (ESTs), and genomic region related to the single-nucleotide-polymorphism (SNP) exploitation, and simple-sequence repeat (SSR) markers. Molecular markers related to the agronomic traits had been demonstrated among different plant species [40,41,42]. Further, the integration of automatic-sequencing machine, Sanger’s sequencing method, and linked data analyzing software had laid the foundation for improvements in sequencing strategies [37,38]. Since then, sequencing technology has revolutionized consistently from the cottage business to the big production enterprise, which demands a sophisticated and dedicated research setup comprising robots with upgrading artificial intelligence and a strong integration with bioinformatics, database setup, up-to-date chemicals, and instruments [43,44].

### 2.2. Next-Generation Sequencing

In 2007, DNA sequencing covered a marvelous milestone to achieve a great step forward for understanding the genomic composition of an organism after the invention of new sequencing strategies. These strategies are designated as next-generation sequencing (NGS) using the high-throughput sequencing methods [43]. In this way, researchers can obtain billions of sequenced DNA nucleotides simultaneously under millions of self-directed chemical reactions by decoding a specific target with high quality and more detailed coverage of short/long sequenced reads of plant species with time-saving and low expense costs [43]. NGS has also been designated as deep sequencing, high-throughput sequencing, or massively parallel sequencing [45,46].

Thus, NGS might require one or two devices or machine deployments to obtain the sequence data during an experiment. It is flexible due to the lack of demand for the precloned DNA region and highly competitive for conducting the genomic data interpretation in contrast to the microarray strategy that depends on tailored arrays of a subject [47]. NGS forums can create genome sequences using the libraries that were constructed by fragmented as well as adapter-attached RNA/DNA/amplicon. It is also better than the conventional vector-constructed approach of cloning and resulting in avoiding or minimizing impurities that appeared due to cloned-DNA sequences under genome sequencing projects [48,49]. Particularly, sequencing strategies follow an ordinary workflow irrespective of a sequencing research forum: such as, (i) library construction using the nucleic acid, (ii) running sequencing machine and aggregate sequenced data, and (iii) finally making data interpretation by bioinformatics or software. Library construction during NGS is a vital step to define sequencing technologies on the basis of the chemical composition of (i) synthesis reaction system, (ii) single-molecule long read, and (iii) ligation chemistry [22,50]. Herein, we briefly described the short-read sequencing and long-read sequencing, which are also known as the second-generation sequencing (SGS) and third-generation sequencing (TGS), respectively.

#### 2.2.1. Second-Generation Sequencing

SGS-short-read forums depend on the construction type of the nucleic acid libraries generated by integrating the DNA strings with the help of adaptors and/or linkers under a ligation reaction. Hence, these DNA regions are not inserted or cloned into a vector or host cells before obtaining decoding sequence data [51,52]. Especially in plant species, the commonly utilized SGS-short-read-associated NGS technologies/models are; (i) Roche 454 (pyrosequencing), (ii) Illumina (Solexa) such as HiSeq and MiSeq methods of sequencing, (iii) oligonucleotide-ligation and detection (SOLiD) sequencing, (iv) BGI Retrovolocity strategy for DNA-nanoball sequencing, and Ion-torrent sequencing. All these formal NGS forums have advantages and disadvantages [53,54,55,56]. So far, several plant species have been sequenced using SGS research forums. For example, the genetic information relating to the several model plants such as *Arabidopsis thaliana*, rice (*Oryza sativa*), maize (*Zea mays*), and papaya (*Carica papaya*) was generated using NGS [40,50]. The genome data can also be accessed by online websites, e.g., https://plabipd.de/index.ep, http://planttfdb.gao-lab.org/, https://phytozome-next.jgi.doe.gov/, http://www.bamboogdb.org/#/, https://www.ncbi.nlm.nih.gov/, etc., which are the edible, medicinal, ornamental, and so on.

The trademark of NGS is more turnout, with countless reactions as compared to the Sanger’s sequencing (FGS), as well as the clonal sequencing. Sample multiplexing in SGS forums can remarkably decrease the cost per sample. NGS also has the potential to overcome the problem of sequencing the haploid fragments, which was a serious problem during Sanger’s sequencing. Until the SGS-short-read has a wide range of applications and dominates the present sequencing market. Many bioinformatic tools are programmed according to the SGS-short-read data analysis and considered more accurate as compared to the TGS-long-read sequencing. Major constraints of the SGS-short-read are; (i) long running times, (ii) generation of de novo assembly is difficult, (iii) structural variations, (iv) the determination of a true isoform of a transcript which is also difficult, (v) haplotype phasing, and (vi) being unable to sequence long fragments of DNA.

#### 2.2.2. Third-Generation Sequencing

TGS-long-read forums have the potential to generate 5 kb (kilobases) to 30 kb read lengths. Previously the longest read-length using the TGS forum is 2 Gb (gigabase pairs) [21]. Thus, the TGS-long-read technology can sequence the single molecule to produce a considerable overlapping read-length for sequence assembly by avoiding the amplification bias [57]. Scientists encountered a persistent problem in dealing with polyploidy genomes of crop plants due to the extensive DNA sequence repetition, a huge genome size to create an assembly of a long chromosome through short-DNA regions. The resulting sequenced DNA data are unable to be mapped according to their genomic or/and chromosome positions [58,59,60]. The above-described reasons created more curiosity among researchers and paved the way for the invention of TGS technologies of NGS. After that, it was common practice to obtain the sequence of a single molecule via TGS-long-read technology that can create the long-DNA sequences or reads and/or scaffolds to encompass the whole chromosome or even genome of an organism. TGS-long-read forum linked methods are (i) DNA dilution constructed, (ii) optical mapping, and (iii) chromosomal-conformation arrest technologies. TGS-long-read research forums are (i) the single-molecule real-time (SMART) sequenced data generated by Pacific-Bioscience, (ii) nanopore sequenced data via Oxford-Nanopore platform (such as MinION and PromethION), (iii) Helico-sequenced data by a genetic analysis system (GAS), and (iv) electron microscopy to generate TGS-long-read data [61,62,63,64]. Currently, TGS-long-read technologies are rapidly taking the place of the SGS-short-read technologies due to the more efficiency of the sequenced data and very low cost of consumption in contrast to the past DNA sequencing technologies. The detailed description of each method, model, and cost of consumption per sample have been reviewed elsewhere [21,65,66,67,68].

#### 2.2.3. Challenges and Limitations of SGS and TGS Forums

The plant itself may cause hurdles for producing a continuous good quality assembly of the genome due to the intrinsic factor(s) (IF). IF can be high heterozygosity, whole-genome duplication (WGD), and polyploidy episodes in organisms under changing climate [69]. The projects relating to the SGS and TGS were executed, numerous polymorphic molecular markers consisting of SNPs were determined, while swiftly creating de-novo genome maps, genotyping-by-sequencing, and transcriptomes, which help to assess the genetic diversity and investigate the traits in plants that can be domesticated [15,49,62,70]. Several isoforms of the transcripts may result from alternative splicing that leads to compositional and functional modifications in protein [71,72]. For example, SGS-short-reads forums possess inherent read-length restrictions that can cause positional genetic information loss and may also undervalue the diversity of an isoform of the transcript [68,73]. Transcripts-isoforms, which possess a different starting transcriptional site and respective RNA processing forms, may require extensive bioinformatic work to precisely process SGS-short-reads into the complete whole-length transcripts [74]. The SGS-short-read technology may be unable to generate the whole-transcriptome annotation due to the WGD event that produces highly similar isoforms of transcripts [74]. While the quantification and identification of transcripts can be possible using SGS-short-read (RNA sequencing) by transcriptome mapping, the novel isoforms cannot be discovered because of the spanning fragments of a transcript [75]. Another challenge of the SGS-short-read RNA sequencing during workflow is the RNA conversion to cDNA that can possibly introduce several library constructions linked biases, i.e., (i) reverse transcription and (ii) amplification and sequence target bias (GC contents) [76]. All the above limitations can be managed using the TGS-long-read technology that can create high-quality whole-length transcripts using a single-RNA molecule by lower coverage-depth. The whole-transcriptome annotation may require full-length sequences, which can be generated by TGS-long-read technology [74,77]. Extrinsic factors (EF) that can affect TGS-long-read are (i) poor quality sample preparations, (ii) sequencing technology (read-length, sequencing coverage, and depth), and (iii) assembly avenues. It also has the potential to resolve the problem of isoform determinations from the long reads or the whole transcripts.

## 3. NGS and Its Promising Aspects

**Figure 3:**
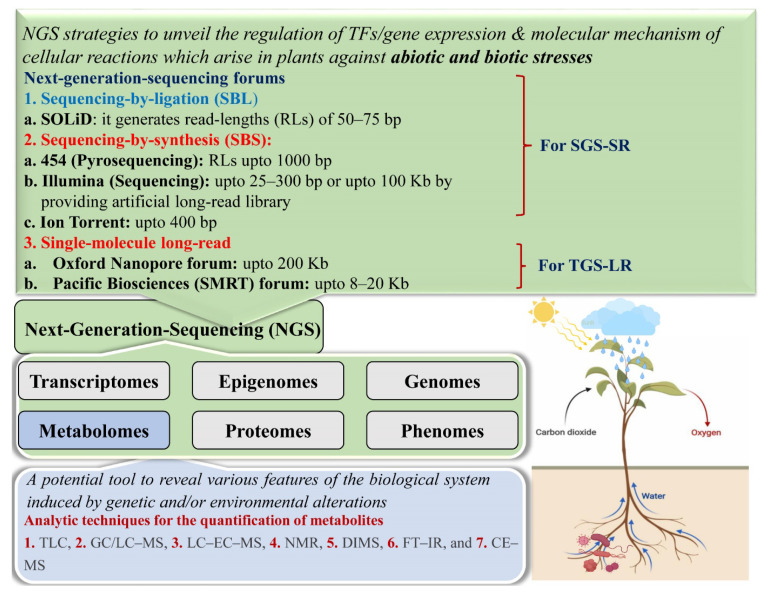
Sequencing forums/technologies, metabolome data generating methods, and plant growth under stressors. In the figure, second-generation sequencing short-read forum—SGS-SR; third-generation sequencing long-read forum—TGS-LR; single-molecule real-time sequencing—SMART; thin-layer chromatography—TLC; gas/liquid chromatography mass spectrometry—GC/LC-MS; LC-electrochemistry-MS—LC-EC-MS; nuclear magnetic resonance—NMR; direct infusion mass spectrometry—DIMS; Fourier-transform infrared—FT-IR; capillary electrophoresis-LC-MS—CE-MS.

Sequencing technologies have always been the foundation of genomics, and during the last 20 years, whole genome or draft of several plant species has been characterized after sequencing or refining by resequencing through various NGS forums/technologies (Figure 3).

### Significance of the NGS

Progress in the NGS technologies has delivered a wide range of research forums with enriched genomic information of the plant species by decoding more complex genomes, e.g., maize, barley, pea, cotton (allotetraploid), wheat (hexaploid), and sugarcane (octoploid), etc., producing long-reads rather than short-reads with limited decoding facts [78]. This progress has also saved time and the cost of expenses and upgraded genome assemblies, leading to normally managing WGS (whole-genome-wide sequencing) tasks using NGS technologies [79].

Currently, it is feasible to generate the high-quality reference genome sequences (RGS) of a plant [65,79]. Another unique advantage of sequencing is to explore the biological niche, local abundance of a species, orphan, or minor crops, which are crucial for national or international ecosystems and participate in the food system. Moreover, the relative of the major crops such as past and current genetic diversity, including wild types, have a special status and genetic information (Table 1). Now, plant scientists can access this genetic information with more detail to find the possibilities or solutions of the current ongoing problems among domesticated plants [66,67]. This acquired knowledge could be more useful in developing climate-resilient crops in the future.

Additionally, NGS has the potential to generate massive data to dissect the novel genes or fragments. Expression profiles among various parts or organs of a plant are determined using NGS to facilitate more specific improvement in plants. For example, the identified genomic maps or regions can be used in developing the potential marker under marker-assisted breeding. A determined expression pattern also helps in uncovering the molecular regulatory processes in a plant under certain stress or normal environments [80]. Thus, NGS provides a research forum along with improving analyzing tools/software or methods [81,82,83] to understand the evolutionary aspects and functions of plants that are taking place under normal or stressed environments by conducting a detailed characterization of a desirable gene or fragment to reveal the complex regulatory mechanism.

## 4. NGS Can Promote Sustainable Crop Production

NGS technologies have now been applied at a large scale in searching for the genetic resources that can pave the path and promise to eradicate food hunger across the world by helping produce or improve crop yield to gain self-sufficiency in food resources. These technologies are creating the genome recourses, improving specificity and efficiency in predicting and designing targeted ESTs, SSR, and SNP markers, genome editing or gene engineering strategies in plants to attain sustainability in production by accurately determining the cause of an appeared trait and/or phenotype under harsh environments [70,84].

### 4.1. Exploiting the Molecular Markers, Genetic Maps, and Phylogenetic Relationships Using NGS Technologies

The genome of an organism retains altering nucleic acid sequences, which could control the phenome or a specific necessary feature as behaving molecular markers, e.g., SNPs are naturally existing several nucleotide or point mutations in the genome of a plant species and resulting to enhance genetic manipulation possibilities to fine-tune a desirable character [38]. Before the creation of molecular markers, it was very difficult to construct a library, perform many cloning works, and finally to sequence [85]. Since then, ESTs have been utilized to discover the SNPs and/or SSRs (microsatellite markers) in the genome, but technologies related to the ESTs also need more funds to generate sequenced data with low genetic coverage [86]. Molecular markers are important in exploring variations among various genomes and determining the quality trait loci (QTLs) in plants to reveal plant traits [87]. For example, QTLs responsible for the grain weight and numbers, yield, sugar accretion, flowering induction or timing, contents of the proline, and other stress-related proteins to increase plant resistance against harsh environments are the key agronomic aspects for sustainable crop yield [88].

Previously, many types of research were carried out to create high-density genetic linkage maps to determine the important QTLs associated with agronomic characters, pinpointing, and isolating the key candidate genes, as well as map-associated gene engineering. Breeders faced several problems as genetic maps with very little information related to the QTLs/molecular markers and low-density by laborious molecular work and higher cost of consumption [89,90,91]. Specifying a molecular marker and respective candidate genes for improving the agronomic traits was not easy in crops before the application of SGS technologies. That said, the accumulating data related to the molecular markers (e.g., in rice QTLs related to the tiller number, etc.) are useful for drawing genetic maps in plant species [91]. SGS technologies have been utilized to generate transcriptome data in various crops [92,93]. Now, researchers can define a particular fragment and/or molecular markers (SNPs and SSRs) by selecting the region(s) of a candidate gene or genome to improve agronomic features of the crop [94]. NGS technologies have transformed the identifications and established the genetic maps by interpreting data associated with the SNPs, SSRs, and QTLs by conducting genome-wide integrated investigations in plant species [95].

Importantly, NGS technologies provide a delightful research forum for scientists to reveal many markers by improving genetic maps with more information about agronomic traits. In this way, these makers participate in genomic selection (GS) for stress-related traits in crops. The genetic tools that have been employed by several plant breeders remain vital for finding the solution of the causes that are drastically influencing plant health, e.g., biotic and abiotic stressors, climate change, etc. [20,96,97,98,99]. Furthermore, these genetic tools can also assist scientists to boost plant yield by determining the desirable traits in crops for improvement. Now, researchers have access to the unique treasure of genome information that can display an important method of genetic manipulation to enhance crop production, after the invention of NGS. For example, genome-assisted breeding (GAB) is forecasted to permit precise and efficient plant breeding to create superior quality cultivars, promoting crop sustainability [80]. Most recently, NGS technologies are fast, less expensive, and have the capacity to sequence many samples within a short time [55]. These salient features promote genotyping technologies such as GAB, marker-assisted selection (MAS; to associate a marker with an appeared trait in a plant), and breeding-assisted genomics (BAG) in an ultrahigh-throughput manner by NGS to determine several molecular markers [17,24,28,86,87]. For example, transcriptome data were generated using NGS technology in lentils and recruited 376 out of 50,960 SNPs, which represent potential targets to control plant traits, e.g., resistance against ascochyta blight, flowering time and color, pigmentation of the stem, seed coat and size, etc. [100,101]. These study outcomes were utilized to construct high-density molecular maps and other markers such as SSRs and ISSR. By analyzing NGS-based transcriptome data, the average space and total coverage distance were improved among two molecular markers, such as 1.11 cM [101,102].

NGS constructed markers permitted a comprehensive phylogenetic genetic analysis of intraspecies and/or interspecies and estimated the divergence time of plants following the wild types and cultivated landraces among various geographic backgrounds. For example, the speciation divergence gaps among *L. culinaris* and *M. truncatula,* as well as *L. ervoides* and *L. culinaris*, were 38 million years ago (MYA) and 0.0677 MYA, respectively [103,104]. Genotyping based on the NGS data revealed geographic distribution and gene pool associated with a specific trait that helps understand the postdomestication pattern and assortment of the improved current morphology of the plant cultivars or species [70,105]. Such outcomes of the past studies laid the foundation for determining the diverse and suitable plant types to perform hybridization during breeding projects to improve genetic resources [106]. Precise divergence analysis can be improved more by generating genome data through NGS technologies in the future. The development in markers (SNPs/SSRs) using NGS could allow the tremendous advancement in designing a plant fingerprinting and forensic science, GS, evolutionary studies, phylogenetic networks, gene flow, genetic maps, etc. [107,108,109,110,111,112,113,114,115,116]. Furthermore, the generation of WGS information of a plant species can lead to more development associated the maker breeding among no-reference available plants.

The noticeable limits to crop breeding evolvement are very slow genetic progress using the crossing, multifaceted characters, and ignored minor or clash crops, which were affected due to the lack of reference genome and/or genetic information until the commencement of NGS technologies. Developing genome data or databases and integrating with the developing analytic tools participate in technology advancement to augment the understanding of genetic resources to respond against several environments by adjusting multi-agronomic traits. Nowadays, formulating quick and precise genotyping strategies to link genome information with phenomes is the pivotal aspect of desirable genetic improvements with the normal affordable expenditure of high-throughput accurate phenotyping. Hence, NGS technologies fuel up the phenotyping strategies with more accuracy to explore genetic or heritable variations in plants under varying environments and decrease the cost of traits’ determinations. More efficiency in NGS technologies is achieved through artificial-intelligence-based robotic ways, standardizing protocols for screening, and launching phenotyping centers for biotic (hotspots for the disease spreading pathogens, insects, pests, etc.) and abiotic (heat waves, drought, land erosion, salinity, and nutrient uptake efficiency) stressors [49,105,116,117,118,119], which are really important factors in formulating precise phenome system to dissect the genomics of quantifiable traits. In the future, portable devices integrated with advanced technologies (ATech) can promote sustainable crop production.

### 4.2. Creating the Pan- or Super-Pan-Genome Based on NGS Technology

Precise genetic manipulation requires more effects to analyze the genomic variations of a population to choose a suitable novel gene to improve agronomic traits and help dissect evolutionary relationships between the species. Researchers cannot only rely on the reference genome of a plant to explore the genetic variations and determine the most suitable population for cultivation. Additionally, sequence coverage or quality of the assembled and annotated reference genomes can also allow the comprehensive understanding of a genome. Generally, the pan-genome describes the genome of a plant/species acquired using the comparative analysis of the huge resequenced genomes, usually the genus [15,18,19,29,44,60,115,116,120]. Such genes can be categorized among various fundamental and rudimentary genes. The fundamental genes are conserved crosswise between various plants and designated as the housekeeping that participate in the key cellular functions, which can be ubiquitin (UBQ), β-actin (ACT), α-tubulin (TUA), ribosomal RNA (subunits such as 18S or 26S) elongation factors (EF), and glyceraldehyde-3-phosphate-dehydrogenase (GAPDH), etc., in different plants. These housekeeping genes can serve as an internal control during validating the expression profile of the targeted genes in the plants under an imposed or naturally occurring stress [121,122,123,124,125]. The rudimentary genes determined using the pan-genome can be conserved within the genome of a particular species or just in some members, however not in the whole genus. By contrast, intercontrol acting essential genes are conserved and integrated with several characters that can be adjustable under stressors in plants, e.g., tolerance to stressors, the activity of the antioxidants and receptors, signal transduction, gene regulation, etc. [126,127,128]. These genes actively shape the diversity of a species and aid in the fast evolution among plants to cope with stressors [129]. Now, targeted resequencing of particular tissues of plants such as mitochondria, plastids (whole plastomes), or even a few regions permits one to establish more precise phylogenetic relationships at the species or subspecies level to reveal genetic diversity and understand the domestication in crops and find ways to boost yield [130,131].

In some plants, high homozygosity and heterozygosity among the candidate genes creates more genetic variations by influencing molecular markers. Therefore, the idea of pan-genome relies on catching variations within or among genomes of different or same species by knowing the genomic structure of the genes [38,116,128]. The NGS has made possible the sequencing or resequencing of many accessions that indicate a plant or species to display structural modifications or variations, varying copy numbers, and including many alterations such as interchromosomal and/or intrachromosomal re-arrangements, transversion, and inversions [43,124,130]. Progress in pan-genome analysis can show the conserved or nonconserved regions between many accessions of a planting species, whereas genomic variations of the accessions belonging to a specific species can be examined by analyzing the super pan-genome [132]. Importantly, super-pan-genome analysis of wild-type plants can present the novel treasure of information relating to the genomic structural variation that can be deployed to mend the agronomic traits in crops by exploring the whole genome of a genus [92]. Both pan- and super-pan-genomes can allow scientists to enhance crop production by creating climate-tolerant plants.

### 4.3. Sustainability by Exploiting the World Genetic Resources

Globally, genetic resources or gene banks are important assets with broad nutritional prospects and safeguarding food security by preserving the vital information that can be utilized to tackle an outbreak of the disease in plants and/or determining the keys genetic factors, which can permit us to create harsh-environment-resilient crops. These genetic resources consist of primitive landraces and plant species, new, extinct, and vanishing plant types, germplasm/lines under breeding programs, wild species of crops and weeds, etc. [16,129], and the articulated evidence about germplasm provision for research purposes by gene stock and other national or international organizations across the world is very limited. Thus, the world’s huge population is deprived of the benefits of the latest innovations relating to the genomic fields due to slow development in developing countries and lack of advanced research bodies.

Although agriculture future relies on crop production within the limited land resources to produce sufficient food to feed the ever-increasing population, it requires more uniformity among genetic materials to cultivate the large-scale area with the same type of crop or species, which can be planted to tackle the increasing events of crop damage by stressors (biotic and abiotic) [8,123]. Even preserved genetic variations can enlighten us to find the cause of an outbreak of disease by exploring the genomic data of the wild or parents’ plants. Many vulnerable, endangered plant species worldwide can show new ways to improve the agriculture future by benefiting from the recent advances in DNA sequencing technologies, especially NGS. Consequently, NGS can also allow scientists to reclassify the bulk of misleading accessions or duplicated by improving identification methods that permit breeders to enhance gene bank management and overcome the common challenges to differentiate the mislabeled genotypes [60,133,134,135,136,137]. The precise assessment of genetic diversity through NGS can lead the researchers to mine the desirable genetic pool, defining the grouping of genetic material, also designating the main or minor target germplasm for research, which could offer the novel cultivars with high resistance to environmental alteration and assist in boosting the sustainability in crop yield.

The generation of sequencing data from the accessions (gene banks) using NGS technologies and the improvements in the bioinformatic tools is the key to determining or choosing the suitable or climate-resilient plants to combat the devastating events forecasted by several researchers [62,83,133]. This sequenced data strengthen more understanding in designing the molecular markers and enhance the accuracy in shaping the allelic variations or detailed genotyping–phenotyping that can improve trait-specific breeding programs. However, gene pools are the treasure that needs to be exploited by NGS around the world, which would develop a clear understanding of inter- or intra-species evolution and reveal the association between wild types and current cultivars that can expand germplasm knowledge to attain sustainable production.

### 4.4. Mining the Novel Genes and Regularity Pathways Using NGS to Generate Transcriptome

Rapid progress in the genomic field has laid the foundation in mining the desirable candidate genes that can help in developing climate-resilient plants, and the utilization of the NGS technologies can lead researchers to adjust or modify the key agronomic traits in crops [62,109,114,135]. The latest developments in technologies have also been employed to obtain sequenced data at a particular stage or time frame of a crop. The acquired data have been submitted or annotated with the determined regularity function to display the information relating to the candidate genes or metabolic pathways integrated with the phenome of a plant. Now, it is relatively easy to find the up-/down-regulated genes or pathways under stress by analyzing the transcriptome generated using the NGS. Studies have revealed several influenced genes or metabolites under abiotic stress at the most critical stage of a crop, e.g., plant reproduction, grain filling, assimilate storage, etc. [138]. Multiple genes or proteins have also been recognized by performing transcriptome analysis such as glycerol-3-phosphate acyltransferase-2 (GPAT2), O-acyltransferase, phosphatidylinositol, or/and phosphatidylcholine transfer protein (SFH13), phosphatidylcholine-diacylglycerol-choline-phosphotransferase (PDCP), plasmodesmata-callose-binding-protein 3 (PDCB), etc. [93]. Under stress, the genes mentioned above were activated, and a few of them encode pyruvate-di-phosphate that participates in shikimate pathways, and this pathway is responsible for producing secondary metabolites in plants under stress [139].

However, such studies reveal more detail about plants’ response to external stimuli by deploying the transcripts to influence the molecular functions and biological and cellular processes that improve the plant tolerance against harsh environments [140]. Synthesized transcriptome data also allow the finding of variations or novel genes among the cultivated cultivars or reference genome of a plant to know altering genetic factors and metabolite under stress. Transcriptome generated by NGS has been employed to unveil the role of genetic material (susceptible and resistant) to resist disease by proving the candidate genes, e.g., *NPR1*, *NPR2*, *calcium-transporting ATPase* (*CT-ATPase*), *glutamate-receptor 3.2*, etc., and many genes related to the phytohormones (jasmonic acid, abscisic acid, and gibberellic acid) that participate in augmenting the plant immunity against pathogen attack [141,142]. Thus, such investigations can be utilized for the genetic manipulation of the desirable crops or plants to create climate-resilient plants that could withstand adverse growth conditions to meet future demands of more food.

## 5. Role of Metabolomics in the Sustainable Crop Production

Metabolome refers to the extensive study of plant-secondary metabolites that regulate various cellular functions in the alive system. This field designates the wide-range set of plant metabolites produced through metabolic pathways in the plant [143,144,145]. Metabolome studies have been largely implicated in the genomic field, which allows us to exploit various phases of the ongoing biomolecular and physiological alteration triggered by climate-change or genetic unrest [146]. Metabolic modifications are directly suggested as the postgenomic changes among plants and facilitate determining the plant phenome or phenotype. Thus, the metabolites are becoming the emerging and the most reliable tools in the plants to unravel stress tolerance or resistance mechanism [147]. For example, respiratory amino acids such as glycine and serine, branched-chain amino-acids (BCAAs), and the few intermediates of the tricarboxylic acid cycle, are found to be assembled in various plants such as barley (*Hordeum vulgare*), (*Oryza sativa*), and *Arabidopsis thaliana* in response to harsh conditions [148,149,150]. Similarly, alterations among levels or numbers of proteins or metabolites such as proline, glycine-butane, tryptophan, phenolic, organic acids, sulpher-responsible metabolites including methionine, cysteine, and glutathione as well as phytohormones (Table 2) have also been affected in plants under stress [151,152,153,154].

Generation of knowledge about specific metabolites related to the critical stage or varying time points to examine the stress response can permit precise genetic manipulation by targeting the particular transcripts in a plant. However, NGS technologies have appeared to be promising breeding tools to describe the regulatory mechanisms and/or cellular reactions against the environmental stimuli that can be the biotic and abiotic stress [108,153]. Moreover, the association between the NGS and metabolomics has enhanced the forecasting abilities of the researchers to find the preliminary metabolic co-networks using the sequenced data of an organism. In this way, the fabricated information generated using NGS technologies and quantification (Figure 3) of the targeted metabolites helps create the most suitable strategy with more precision to increase crop yield. These technologies can speed up the genetic manipulation programs or projects to create climate-resilient smart crops that can be a source of nutritious food as well as the key to eradicating hunger across the world by safeguarding food security [143]. There is no doubt that phenotypic or phenome-directed genetic manipulation has been witnessed to improve the performance of a crop by performing metabolic engineering in the same manner as the genomic fields have demonstrated the substantial participation in accomplishing more advances in genomic research gains [144,145,182].

## 6. Diagnosis and Monitoring of the Disease-Causing Pathogens Using NGS and Metabolites

Multiple pathogens are casual disease agents including the vast diversity of bacteria, protozoa, mollicutes, fungi, virods, and viruses [183,184]. Researchers have employed several molecular techniques to diagnose and monitor the aforementioned pathogens to design control strategies and improve crop production. Previous techniques were not efficient enough to determine the minute residues of an organism, especially viruses and evolving pathogens. Then, the advent of genomic science assisted scientists to reveal the genetic composition of crops and the disease-causing pathogens and provided the key clues to monitor plant and pathogen interaction for crop improvements by employing the NGS and metabolomics to determine the host genetic factors that the disease-causing agent engineers during attack or infection and the ability to gain defense through reprogramming the genetic makeup [185]. For example, the sugarcane mosaic virus (SCMV) is a major threat to the maize-cultivating farming community in China’s maize-cultivating farming community; it was revealed by analyzing transcriptomic data that SCMV actively participates in downregulating the photosynthesis-responsible genes, leading to the phenotype of chlorotic lesions [186].

Recent NGS-based deep-sequencing technologies generated reliable sequenced genomes to carry out genomic analysis using the developed bioinformatic resources to improve disease control approaches by accurately diagnosing plant pathogens [112,155,156,157]. These ATech can be employed to produce metagenomics and estimate the infecting or developing microbial population in a crop [187]. Moreover, exploring the small RNA (sRNA) families such as interfering RNAs (siRNAs) might be utilized to perform identification as well as reconstruction of multiple virus genome (DNA or/and RNA) and belonging microvariants using the latest bioinformatics approaches. NGS technologies are also applicable to find harboring pathogens by insect vectors, crop certification, and quarantine programs by diagnosis techniques. Nowadays, it is possible to detect plant metabolites (Table 2), e.g., flavonoids, cyanogenic glycosides, benzoxazinoids, saponins, terpenes, and terpenoids, which are produced under a pathogen attack in various crops such as rice, maize, rye, barley, oat, millet, sorghum, etc. [146,151,153,154]. Therefore, a combined strategy based on the genomic and metabolomics analysis can stabilize the declining plant yield across the world.

## 7. Integration between the Transcriptome and Metabolome to Achieve Crop Sustainability

Integrated networks have been established by analyzing the transcriptome and metabolome data to pave the path for a more accurate engineering of the metabolites through genomics in plants [188,189]. This aspect enlightens us to reveal the importance of metabolites and transcripts in plants to acquire stress tolerance and provide more evidence to develop a comprehensive strategy to increase crop yield. Both metabolome and transcriptome also have the potential to show the key metabolites and/or cellular processes that can affect the plant architecture and biomass production and participate in plant adjustments by regulating the physiological state of an organism [146]. Furthermore, recent advances in technologies have displayed novel metabolic networks and have pinpointed the key regulatory genes by dissecting the genetic of transgene lines or mutants [139,190,191].

In addition, these techniques display the role of a gene to influence the metabolic pathways and unmask the underlying sophisticated mechanisms and coordination that are established among various pathways, which are hardly obtainable through conventional techniques such as microarray [192]. Successfully, these techniques have been employed to conduct metabolic engineering in various crops, e.g., anthocyanin production altered in tomato, and the nutritional reserved of rice’s endosperm has also improved by increasing the accumulation of β-carotene [193]. The availability of advanced genetic manipulation technology such as CRISPR/Cas9 may improve the metabolome and transcriptome related studies in plants. CRISPR/Cas9 has been used to create the germplasm with superior quality and traits in plants [194,195,196,197]. Moreover, development in bioinformatics fields can participate in a better assembly of the WGS of a plant. In this way, the strategies to reveal the genome-wide genetic alterations and the genotyping approaches will be improved in a cost-effective manner. Then, researchers will be able to precisely build integration among various technologies to bring a revolution in plant breeding and/or engineering. These efforts may facilitate attaining sustainability objectives without degrading our environment to feed the expanding population of the world.

## 8. Understanding the Bamboos’ Tolerance Using NGS and Metabolome

Climate variations are badly affecting food security in all continents. According to a prediction, more than 50% of agriculture losses occurred due to stressors that are considered a big future challenge to fulfill the demands of the ever-increasing population by producing enough resources [198,199]. Forests have significant potential to aid the mitigation of anthropogenic activities of climate disturbance and offer several co-benefits by building a healthy society [200]. Unfortunately, prevalent climate-persuaded forest die-off has also been estimated among forests worldwide. It forms an unsafe carbon-cycle feedback, discharging a huge quantity of stored carbon from the forest ecosystem to the atmosphere, as well as declining the volume of carbon sink for future forests. Plant mortality occasions have been witnessed across the world during the last decades due to climate change [118,201]. The direct impacts of climate on plants, such as drought, salt, high-temperature incidences, including other affecting agents such as wildfire and pathogen/insect epidemics, are vulnerable to climate alterations and have a major influence on forests [117,202,203]. Therefore, forests of bamboos have a significant role in sustainable agriculture production. Especially, *Phyllostachys edulis* belongs to a novel forest resource with an extraordinarily fast growth, monopodial-rhizome network, and immense significance in the ecosystem, restoring degraded lands, being a source of income for ~2.5 billion people, a raw material for industrial use, and a fighting weapon against climate alterations in several American, African, and Asian countries [204,205]. Internationally, bamboo trade accounts for at least $2.5 billion per year, and a consistent increase in trade has been observed [204]. Forests of moso bamboo cover approximately 73.8% of the total bamboo forest area in China and have a great cultural impact [206]. Bamboo is utilized in decorating recreational places, manufacturing furniture, musical instruments, houses, and food dishes, with the shoots being especially very popular as nutrient-enriched tasty food (bamboo shoots) in Southern China [207,208].

**Figure 4:**
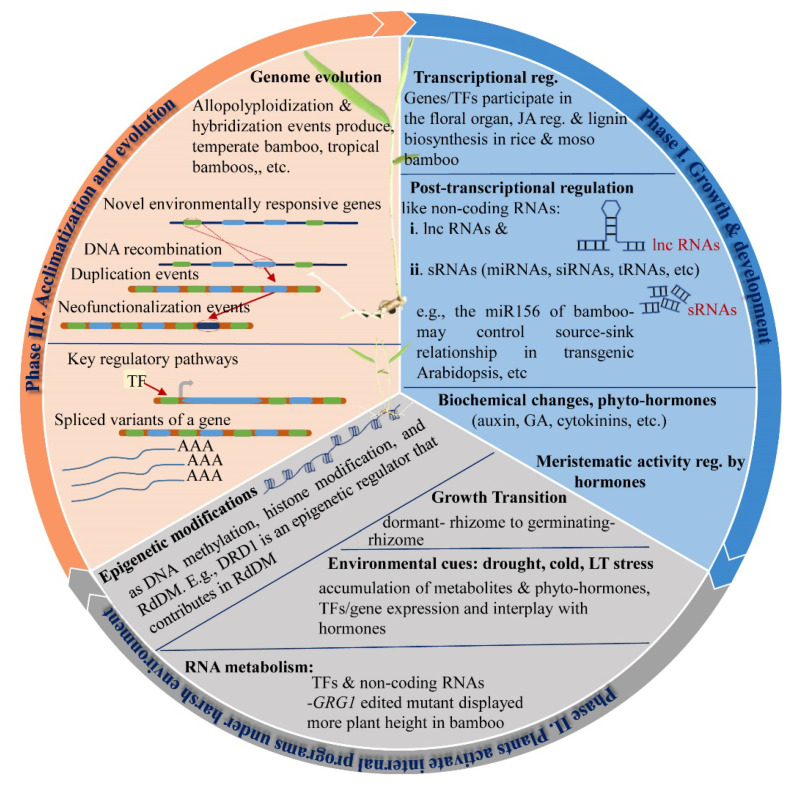
Various Growth and development phases and stress tolerance mechanism in bamboos. Different genes/TFs and non-coding RNAs participate in functional regulation during the growth and development phase I to perform the specific or multiple roles. The biochemical alteration also defines the growth transition (e.g., culm development under an altering gradient of hormones as the GA, IAA, ABA, zeatin (ZT). Genes-responsible for the lignin (PvNST1/2–1, PvC3H-2/3, PvC4H-2/4, PvCADs, PvCCR-2/4, PvHCT-2/5/8, PvPAL-2/4/6) and JA (PvOPR2, PvPEX5, PvJAZ-4) synthesis; early flowering (PeMADS2), etc. During phase II, plants regulate internal adjustments to cope with stressors such as hormone alterations (e.g., rhizome generates new shoot under an altering gradient of hormones), RNA metabolism, epigenetic modifications, and accumulation of various plant metabolites. Phase III is considered acclimatization and evolution; many evolution events take place during evolving plants to produce multiple copies of transcripts as compared to the ancestral donors to flourish new generations of plants under consistent overwhelming environments. Abbreviations in figure: regulation—reg.; jasmonic acid—JA; gibberellic acid—GA; indole-3-acetic acid—IAA; abscisic acid—ABA; zeatin—ZT; long-noncoding ribonucleic acid—lnc RNAs, small noncoding RNAs—sRNAs; short interfering RNAs—siRNAs; microRNAs—miRNAs; transfer RNAs—tRNAs; RNA-directed DNA methylation—RdDM; gibberellic acid—GA; indole-3-acetic acid—IAA; abscisic acid—ABA; zeatin—ZT; low-temperature—LT; peroxidase—POD; phenylalanine-ammonia-lyase- PAL; and 4-coumarate responsive ligase—4CL.

Recently, high-quality genome data of moso bamboo (*Phyllostachys edulis*) were reported; they can be the key to identifying the various TFs/genes having more similarity with the reported Arabidopsis’s genes. These identified targets in bamboo may perform similar or different functions upon functional characterization in homologous or heterologous systems. More duplication events are also found in bamboo’s genome to display more copies of a gene than Arabidopsis. For example, NAC TFs related to the fiber development in rice, Arabidopsis, and moso bamboo are different in numbers such as 6, 8, and 16, respectively. Similarly, the homologous genes of *OsNAP* and *OsNAC10* are six and three in moso bamboo [209,210,211]. It is also noted that moso bamboo presented more duplication events during genome evolution than rice; many genes display a specific function such as floral organ development-related genes in bamboo, and their respective high homology genes in rice, lignin, jasmonic acid, and stress-responsive genes [212] have been reported in bamboo (Figure 4). Bamboo growth requires indispensable energy resources and the integrity of the cell wall to perform normal physiochemical processes for maintaining bamboo’s fast growth (Figure 4) [211]. Furthermore, abiotic stresses create a serious impact on bamboo growth and development [213]. Access to the moso bamboo genome [210,211] provides a chance to many researchers for genome-wide classifications of TFs such as aquaporin, AAAP, UBP, IQD, HD-Zip, Hexokinase, Aux/IAA and ARF, NAC, PeUGE, HSF, and CONSTANS-like in moso bamboo [188,214,215,216,217,218,219,220,221,222,223].

**Figure 5:**
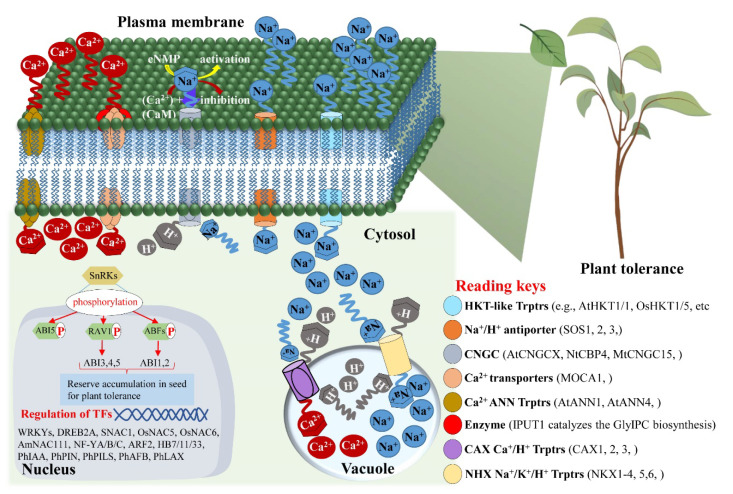
Regulation of plant tolerance through transporters and/or transcription factors. Osmatic regulation is attained in plants by opening and closing of channels using transporters (Trptrs) related to the cations (Ca^2+^, Na^+^, and H^+^). Furthermore transcription factors (TFs) play a crucial role in operating plant tolerance (e.g., SnRKs interact with other TFs and/or genes by phosphorylating and activating more genetic factors to help plants build food reverse that can be utilized under the stress condition. In the figure, HKT: high affinity K^+^ transporters; SOS: salt overly sensitive 1; (X): various transporters/genes such as 1, 2, 3, 4, 5, 6, 7, 8, 9, 10, 12, 14, 16, 18, and 20; CNGC: cyclic-nucleotide-gated channels; MOCA1: monocation-induced Ca^2+^ enhancer 1; ANN: Annexin; IPUT: inositol-phosphorylceramide glucuronosyltransferase; GlyIPC: glycosyl-inositol-phosphorylceramide; CA: cation exchanger; NH: Na^+^/H^+^ exchangers; cNMP: cyclic-nucleotide monophosphate; and CaM: calmodulin.

Genome-wide classifications of TFs’ families in moso bamboo have been carried out and have demonstrated a limited molecular characterization in model plants by exogenous gene transfer in rice and Arabidopsis and the expression profile against stresses [224,225,226]. The expression of *PeLAC* was high in stem and its promoter sequence contains ABRE cisregulatory element that responds to ABA and GA treatments by upregulating and downregulating the transcript of *PeLAC*, respectively [227]. Stress tolerance in bamboo (*Phyllostachys edulis*) was investigated by characterizing the tonoplast-intrinsic proteins (TIPs). Results suggested that PeTIPs may improve abiotic stress in bamboo [228]. *PheWRKY86* coordinates with NCED1 by binding the W-box within the promoter region and improve stress tolerance in transgenic rice and Arabidopsis [229]. Osmotic adjustment participates in plant stress tolerance and development mechanism; e.g., Ca^2+^ translocation in and out from the vacuole, cell wall, and intercellular compartments regulate the development of phloem ganglion in *P. edulis* during the active early growth phase. Later, at the maturation stage of phloem, more accumulation of vacuolar Ca^2+^ was observed as compared to mature cells of cytoplast. Results suggested the important role of Ca^2+^ in generating cells and the osmatic actions of phloem ganglion in *P. edulis* [230]. *PeNAC-1* has been suggested to regulate Na^+^ across the cellular membrane and may affect the Na^+^/K^+^ homeostasis [206] in the heterologous systems as several other phytohormones and ion transporters perform the function in plants under normal and stressed condition plants (Figure 5) [231,232]. Many TFs/genes and transporters have been identified in bamboo, e.g., genes responsive to multiple hormones (cytokinins, gibberellic acid, jasmonic acid, abscisic acid, ethylene, and auxin), and auxin biosynthesis-related transporters (PhIAA, PhPIN, PhPILS, PhAFB, PhLAX, etc.) have been identified in bamboo [233].

The accumulation of metabolites also helps plants to encounter stress conditions. An increase in anthocyanin contents was examined in Ma bamboo in the overexpressing leaf-color-related gene, which improved stress resilience against cold and drought conditions by enhancing stress-related antioxidant activity [225]. Very limited research is available on the heterologous system investigations of genes retrieved using transcriptome data of bamboo [228,234,235]. For example, the high expression of *PeUGE* was validated in shoots [228]. Later on, *PeUGE* investigations in overexpressing Arabidopsis plants suggested it regulates cell wall biosynthesis and improves stress tolerance. A significant expression alteration of *Phehdz1* was induced by drought, salinity, and ABA treatments, but the high expression was in roots. Overexpressing transgene rice (OE-Phehdz1) displayed improved DS tolerance and altered secondary metabolism [225]. The lignification of tissues in bamboo is a unique character that contains monolignol glucosides (i.e., syringin, coniferin, p-glucocoumaryl alcohol, and guaiacyl) that have been examined as the key storage components of lignin precursors, which are transported to the outer cells [236,237]. ATP-binding cassette transporters have been suggested to regulate translocation of lignifying agents using vascular cation transporters by establishing a gradient across and within cellular membrane and cytoplasm [237] that needs to be exploited in detail.

Furthermore, investigations related to cutin and suberin biosynthesis in bamboo can promote a clear understanding of the stress tolerance mechanism in bamboo [238,239,240,241,242,243]. Despite advances in technology, the enhancement of agricultural resources requires consistent improvements in genome editing and metabolome technologies. During the last decade, several scientists used the CRISPR/Cas9 genome editing tool to obtain desirable traits in plant species [244]. However, very limited research revealed successful genome editing by CRISPR/Cas9 after selecting a desirable gene in ma bamboo [245]. Reports have been demonstrated that in ma bamboo, plantlets can be regenerated by callus induction in germinating embryos [246]. This literature review supports further development in gaining a better understanding through NGS and metabolome knowledge to manipulate bamboo’s genome to meet the future demands of sustainable forest resources across the globe.

## 9. Concluding Remarks and Promising Future Perspectives

Currently, crop production is unable to feed the growing world population due to deteriorating natural resources or mismanagement of the available genetic resources rather than other agriculture challenges. Under a perfect scenario, the pure wild-type progenitor individuals are necessary for the marking out crop domestication information and the clear identification of crop or a plant desirable sweep fragments or genes among the genome. Therefore, the improvements of genetic makeup are heralded as the prominent aspect to boost crop yield, generate climate-resilient plants/crops, and enhance nutritional value. The application of advanced bioinformatics and genetic tools grasp the considerable assurance for more agriculture output, increased livelihoods, and multiple prospects for food security by exploring the potential of the cash and orphan crops.

Improved genome information of plants will allow one to carry out effective and accurate genetic engineering in crops by revealing the mechanism of the most important agronomic traits. However, NGS technologies are evolving rapidly and claim to generate long-length strings of genomic reads, within less time and lower cost per sample or unit. Now, scientists can produce whole-genome assembly, de-novo assembly metagenomics, transcriptome, methylome sequencing, etc. Hence, NGS application is very important in many fields related to agriculture to find targets for genetic manipulation, evolution studies, exploring the bionetworks, and understanding the fundamental principles of functional genomics. Improved molecular markers will speed up breeding programs. Furthermore, metabolome studies are vital for producing knowledge about physiological mechanisms and/or metabolic pathways regulated in a crop under a stress condition to allow plant adaptation against harsh environments, to build integrated networks among genomic and metabolomics and to help the researcher to accomplish enriched information to estimate phenotype and manipulate the agronomic trait by genome and/or metabolite engineering in plants. However, a broad-spectrum application of the super- and pan-genome remains to be exploited to attain more precision in determining the phenotypes in plants related to the agronomic traits.

Particularly, developing countries are lagging to obtain benefits from the advanced technologies (ATech) due to very few investments to establish the state-of-the-art sequencing genomic center and large-scale or even small-scale robotic laboratories and provide services to the farming communities for the pathogen identification to design a better control strategy. Lack of funds for establishing infrastructure, less awareness about advanced technologies, training workshops, or increasing corruption may be the causes of decline in agriculture production. Now, the government and private sectors are actively participating in transfer-technology collaborative programs to disseminate the ATech among farming communities and open Hi-Technology centers to provide services and utilize bioinformatics and biotechnological tools to enhance crop production. Universities are also training students and producing the researchers that can construct NGS libraries and generate sequence data to interpret a better understanding of stress regulation mechanism in a crop. People can visit the online-accessible portal to acquire more information related to crop diseases or pathogens using artificial-intelligence-equipped devices or smart phones due to high-speed internet availability.

Additionally, it is necessary to refine genome assemblies by resequencing reference genomes with advanced NGS technologies and determining metabolites among cultivated and wild plants. Integration among various fields such as transcriptomes, epigenomes, proteomes, phenomes, genomes, and metabolomes, also demands advanced programming language and database creation to build a network that can assist in understanding the molecular mechanism of an appeared phenotype in plants. Bulk data generation requires an automated pipeline or operating systems that can perform routine tasks such as the generation of sequence data and interpretation of the results using the developed build-in bioinformatic tools. Consequently, comprehensive knowledge in ATech will facilitate comparative genomics to explore unmined genomes of plants and improve or find new ways of sustainable crop production.

## Figures and Tables

**Table 1:** Information of the sequenced species provides a source of genetic manipulation and understanding of the domestication process.

Family Name	Organism	GS	NPTs	Online Accessible Links
*Amaranthaceae*	*Beta vulgaris* (sugar beet), spp. vulgaris var. cicla)	604 Mbp	34,521	https://bvseq.boku.ac.at/
	*Suaeda aralocaspica* (shrubby sea-blite)	467 Mbp	29,604	https://www.ncbi.nlm.nih.gov/bioproject/?term=PRJNA428881
*Arecaceae*	*Elaeis guineensis* (African oil palm)	1800 Mbp	25,405	https://www.ncbi.nlm.nih.gov/genome/?term=txid51953[orgn]
	*Phoenis dactylifera* (date palm), an elite variety (Khalas)	605.4 Mbp	~41,660	https://pubmed.ncbi.nlm.nih.gov/23917264/
*Brassicaceae*	*Arabidopsis thaliana* (Arabidopsis)	125 Mbp	~27,025	https://www.arabidopsis.org/ and https://www.nature.com/articles/ng.807
	*Arabidopsis lyrata* (Arabidopsis)	207 Mbp	~32,670	https://www.arabidopsis.org/ and https://www.nature.com/articles/ng.807
	*Capsella rubella* (pink shepherd’s-purse)	134.8 Mbp	~28,447	https://www.nature.com/articles/ng.2669
	*Eruca sativa* (salad rocket)	∼851 Mbp	45,438	https://www.frontiersin.org/articles/10.3389/fpls.2020.525102/full
	*Eutrema salsugineum* (saltwater cress)	241 Mbp	26,531	https://www.frontiersin.org/articles/10.3389/fpls.2013.00046/full
*Cannabaceae*	*Cannabis sativa* (hemp)	808 Mbp	38,828	https://www.nature.com/articles/s41438-020-0295-3
*Cactaceae*	*Carnegiea gigantea* (saguaro)	1.40 GB	28,292	https://www.pnas.org/content/114/45/12003
*Cucurbitaceae*	*Cucumis melo* (musk melon), doubled-haploid line DHL92	375 Mbp	27,427	https://www.pnas.org/content/109/29/11872#abstract-1
	*Cucumis sativus* (cucumber), ‘Chinese long’ inbred line 9930	226.2 Mbp	26,682	https://academic.oup.com/gigascience/article/8/6/giz072/5520540
*Dioscoreaceae*	*Dioscorea rotundata* (Yam)	594 Mbp	26,198	https://bmcbiol.biomedcentral.com/articles/10.1186/s12915-017-0419-x
*Euphorbiaceae*	*Manihot esculenta* (cassava), domesticated KU50	495 Mbp	37,592	https://www.nature.com/articles/ncomms6110#Sec8
*Fabaceae*	Cajanus cajan (pigeon pea)	833.07 Mbp	48,680	https://www.nature.com/articles/nbt.2022
	*Cicer arietinum* (chickpea)	∼738 Mbp	28,269	https://www.nature.com/articles/nbt.2491
	Glycine max (soybean), cultivar Williams 82	969.6 Mbp	46,430	https://www.nature.com/articles/nature08670#Sec9
	*Medicago turncatula* (medick or burclover)	~330 Mbp	50,894	http://europepmc.org/article/MED/24767513
	*Vigna unguiculata* (cowpea)	640.6 Mbp	29,773	https://onlinelibrary.wiley.com/doi/full/10.1111/tpj.14349
*Ginkgoaceae*	*Ginkgo biloba* (ginkgo)	10.61 Gb	41,840	https://gigascience.biomedcentral.com/articles/10.1186/s13742-016-0154-1
*Musaceae*	*Musa acuminata* (Banana) spp. Malaccensis	523 Mbp	36,542	https://www.nature.com/articles/nature11241
*Pinaceae*	*Picea abies* (Norway spruce)	20 GB	28,354	https://www.nature.com/articles/nature12211
*Poaceae*	*Hordeum vulgare* (barley)	5.1 GB	26,159	https://www.nature.com/articles/nature11543
	*Oryza sativa* (rice)	373.2 Mbp	3475	https://www.ncbi.nlm.nih.gov/pmc/articles/PMC5395016/
	*Phyllostachys heterocycla var. pubescens*	2.05 Gb	31,987	https://www.nature.com/articles/ng.2569
	*Phyllostachys edulis*	1.91 GB	51,074	https://academic.oup.com/gigascience/article/7/10/giy115/5092772
	*Raddia distichophylla (Schrad. ex Nees) Chase*	589 Mbp	30,763	https://academic.oup.com/g3journal/article/11/2/jkaa049/6066164
	*Sorghum bicolor* (sorghum), Rio genetic material	729.4 Mbp	35,467	https://bmcgenomics.biomedcentral.com/articles/10.1186/s12864-019-5734-x#Sec6
	*Triticum urartu* (einkorn wheat), accession G1812 (PI428198)	~4.94 GB	34,879	https://www.nature.com/articles/nature11997
	*Zea mays* (maize), B73 inbred maize line	2.3 GB	>32,000	https://pubmed.ncbi.nlm.nih.gov/19965430/
*Salicaceae*	*Populus tricchocarpa* (poplar)	380 Mbp	37,238	https://www.pnas.org/content/115/46/E10970#sec-1
*Solanaceae*	*Capsicum annuum* (pepper)	3.06 GB	34,903	https://www.nature.com/articles/ng.2877#Sec10
	*Nicotiana benthamiana* (tobacco)	3.1 GB	42,855	https://www.biorxiv.org/content/10.1101/373506v2
	*Solanum lycopersicum* (tomato), cv. Heinz 1706	799.09 Mbp	34,384	https://www.biorxiv.org/content/10.1101/2021.05.04.441887v1.full.pdf
	*Solanum tuberosum* (potato)	844 Mbp	39,031	https://www.nature.com/articles/nature10158/
*Arecaceae*	*Elaeis guineensis* (African oil palm)	1.8 GB	~34,802	https://www.nature.com/articles/nature12309
	*Phoenis dactylifera* (date palm), an elite variety (Khalas)	605.4 Mbp	~41,660	https://europepmc.org/article/PMC/3741641
*Rosaceae*	*Prumus persica* (peach)	247.33 Mbp	26,335	https://onlinelibrary.wiley.com/doi/10.1111/tpj.15439?af=R
*Vitaceae*	*Vitis sylvestris* (grape), accession of Sylvestris C1-2	469 Mbp	39,031	https://genomebiology.biomedcentral.com/articles/10.1186/s13059-020-02131-y#Sec2

Genome size—GS; number of predicted transcripts/proteins—NPTs.

**Table 2:** Different plants produce various kinds of plant metabolites at varying developmental stages under stress conditions by regulating primary and/or secondary metabolism.

Plant	E	Stage and Specific Organ	Metabolites	Refs.
*Avena sativa* (oats)	E1	Not specified using grains	PM **: malic, gluconic, and galacturonic acids, fatty acids (FAs), palmitic acid and linoleic acid.	[155]
	E2	Seedling stage (three weeks old) Leaves	PMS **: Ascorbate, aldarate phenylpropanoids.	[156]
*Hordeum vulgare* (barley)	E1	Germination using seeds	PM *: glycero(phospho)lipids, prenol lipids, sterol lipids, methylation. SM *: polyketides.	[157]
	E2	Two-leaf stage seedlings using leaves	PM **: organic acids (OAs), amino acids (AAs), nucleotides, and derivatives. SM *: flavonoids, absiscic acid.	[158]
	E3	Three-leaf stage using leaves	SM **: chlorogenic acids, hydrocinnamic acid derivatives, and hordatines and their glycosides.	[119]
	E4	Three-leaf stage and flag leaf stage using leaves	SM *: flavonoids, hydroxycinnamic acid, phenolics, glycosides, esters, and amides.	[159]
	E5	During grain filling using seeds	PM *: Tricarboxylic acid (TCA), OAs, aldehydes, alcohols, polyols, FAs, carbohydrates, mevalonate. SM **: phenolic compounds, flavonoids.	[10]
	E6	Four weeks old using leaves	PM *: carbohydrates, free AAs, carboxylates, phosphorylated intermediates, antioxidants, carotenoids.	[160]
	E7	1–3 weeks old using leaves and roots	PM **: AAs, sugars, OAs as fumaric acid, malic acid, glyceric acid	[161]
*Oryza sativa* L. (rice)	E1	Flowering and early grain filling stages using leaves, spikelets, seeds	PSM **: isoleucine, 3-cyano-alanine, phenylalanine, spermidine, polyamine, ornithine	[161]
	E2	At reproductive stage using leaves and grains ripe stages	PM **: saturated and unsaturated FAs, AAs, sugars, and OAs.	[162]
	E3	24 months old seeds used	PM **: sugar synthesis related compounds, AAs, free FAs, TCA cycle intermediates.	[163]
	E4	Not specified using grain	PM *: aromatic AAs, carbohydrates, cofactors and vitamins, lipids, oxylipins, nucleotides. SM *: benzenoids.	[164]
	E5	Maturation using mature seed	PM *: carbohydrates, lipids, cofactors, prosthetic groups, electron carriers, nucleotides. SM *: benzenoids.	[165]
	E6	Maturation using mature seed	PM *: carbohydrates and lipids. SM *: α-carotene, β-carotene, and lutein.	[166]
	E7	Six weeks old using leaves	PM *: AAs (arginine, ornithine, citrulline, tyrosine, phenylalanine and lysine), FAs and lipids, glutathione, carbohydrates. SM *: rutin, acetophenone, alkaloids.	[157]
*Setaria italica* (foxtail millet)	E1	60 days using shoots	PM *: fructose, glucose, gluconate, formate, threonine, 4-aminobutyrate, 2-hydroxyvalerate, sarcosine, betaine, choline, isovalerate, acetate, pyruvate, TCA-OAs, and uridine.	[167]
	E2	3–5 leaves stages using leaves	PM *: glycerophospholipids, AAs, OAs. SM: flavonoids, hydroxycinnamic acids, phenolamides, and vitamin-related compounds.	[167]
*Sorghum bicolor* (sorghum)	E1	Four-leaf stage using leaves	PM *: AAs, carboxylic acids, FAs. SM: cyanogenic glycosides, flavonoids, hydroxycinnamic acids, indoles, benzoates, phytohormones, and shikimates.	[168]
	E2	Four-leaf stage using leaves	SM *: 3-Deoxyanthocyanidins, phenolics, flavonoids, phytohormones, luteolinidin, apigeninidin, riboflavin.	[169]
	E3	Around 26 days using roots and leaves	PM *: sugars, sugar alcohols, AAs, and OAs.	[170]
	E4	Four weeks old using grain and biomass	PM **: OAs. SM **: phenylpropanoids.	[146]
*Triticum aestivum* (*wheat)*	E1	NAS using leaves	PM *: sugars, glycolysis and gluconeogenesis intermediates, AAs, nucleic acid precursors, and intermediates. SM *: chorismate, polyamines, L-pipecolate, amino-adipic acid, phenylpropanoids, terpene skeleton, and ubiquinone.	[171]
	E2	Physiological maturity using leaves	PM *: AAs metabolism, sugar alcohols, purine metabolism, glycerolipids, and guanine. SM *: shikimates, anthranilate, absiscic acid.	[172]
	E3	Maturation using matured kernels	PM *: FAs, sugar, nucleic acids and derivatives. SM *: phenolamides, flavonoids, polyphenols, vitamins, OAs, AAs, phytohormones, and derivatives.	[173]
	E4	Not specified using grain	PM *: osmolytes, glycine betaine, choline, and asparagine.	[174]
	E5	Not specified using seeds	PM *: sterols, FAs, long chain FAs derivatives, glycerol (phospho) lipids. SM *: polyketides.	[175]
*Zea mays* (maize)	E1	R6 stage using grains	PM **: sugars, sucrose, glucose, and fructose.	[176]
	E2	Physiological maturity using kernels	PM *: glycolysis, TCA cycle, starch, amino acids. SM: alkaloids, benzenoids, fatty acid and sugar derivatives, flavonoids, phenylpropanoids, and terpenoids.	[177]
	E3	8 months using kernels	PM *: glucose, fructose, sucrose, tocopherol, phytosterol, inositol, asparagine, glutamic acid, pyroglutamic acid.	[178]
	E4	Eight-visible-leaf stage using leaves	PM *: choline, inositol, sugars, raffinose, rhamnose, TCA cycle, AAs, trigonelline, putrescine, quinate, shikimate. SM *: flavonoids, and benzoxazinoids.	[179]
	E5	Seedling stage using entire seedling	PM *: amino acids, lipids, carboxylic acid. SM *: alkaloids, terpenoids, flavonoids, alkaloids, and benzenoids.	[180]
	E6	Physiological maturity using kernels	SM *: flavanones, flavones, anthocyanins, and methoxylated flavonoids.	[181]

E—experiment; **—upregulation/significant contents; *—difference examined as compare to control/mock; primary and secondary metabolism/metabolites—PSM; primary metabolism/metabolites—PM; secondary metabolism/metabolites—SM; not available stage—NAS.
